# Memory Cells in Infection and Autoimmunity: Mechanisms, Functions, and Therapeutic Implications

**DOI:** 10.3390/vaccines13020205

**Published:** 2025-02-19

**Authors:** Shilpi Giri, Lalit Batra

**Affiliations:** 1 Department of Immunology, University of Pittsburgh, Pittsburgh, PA 15213, USA; 2 Center for Predictive Medicine for Biodefence and Emerging Infectious Diseases, School of Medicine, University of Louisville, Louisville, KY 40222, USA; lalit.batra@louisville.edu

**Keywords:** memory T cells, memory B cells, infection, autoimmunity, pathogenic memory responses, therapeutic targeting of memory cells, cytokine inhibitors, inflammation and immune regulation

## Abstract

Memory cells are central to the adaptive immune system’s ability to remember and respond effectively to previously encountered pathogens. While memory cells provide robust protection against infections, they can also contribute to autoimmunity when regulation fails. Here, we review the roles of memory T and B cells in infection and autoimmunity, focusing on their differentiation, activation, effector functions, and underlying regulatory mechanisms. We elaborate on the precise mechanisms by which memory cells contribute to autoimmune diseases, highlighting insights from current research on how pathogenic memory responses are formed and sustained in autoimmunity. Finally, we explore potential therapeutic strategies aimed at modulating memory cells to prevent or treat autoimmune disorders, including B cell-depleting therapies (e.g., Rituximab), T cell-targeting agents (e.g., Abatacept), and cytokine inhibitors (e.g., IL-17 or IL-23 blockers) that are currently used in diseases such as rheumatoid arthritis, multiple sclerosis, and psoriasis.

## 1. Introduction

Memory immune cells are central to the adaptive immune system’s ability to provide long-lasting protection against pathogens while maintaining tolerance to self-antigens. These specialized cells, derived from prior immune responses, retain the capacity to mount rapid and robust responses upon re-exposure to antigens [1]. Beyond their role in infection, memory cells are increasingly recognized for their contributions to the pathogenesis of autoimmune diseases, where their persistence and reactivity can exacerbate self-directed immune attacks. This review explores the multifaceted roles of memory T cells and B cells in both infection and autoimmunity. We examine the functional adaptations that endow memory cells with their distinctive characteristics, such as their enhanced survival, metabolic reprogramming, and rapid recall responses. By contrasting the protective roles of memory cells in infection with their pathological contributions in autoimmunity, we aim to highlight the dual-edged nature of these cells. This synthesis of current knowledge underscores the potential for targeting memory cell dynamics as a therapeutic strategy, either to bolster immune defense or to mitigate autoimmune tissue damage. Through this discussion, we provide a comprehensive framework for understanding the biology of memory immune cells and their implications for health and disease.

## 2. Types of Memory Cells and Their Generation


**Memory Cell Type**

**Characteristics**

**Generation Process**

**References**

**T Cell Memory Subsets**
Central Memory T Cells (TCMs)Express CD62L and CCR7 (lymphoid homing), High proliferative capacity, Delayed effector functionDerived from naïve or effector T cells, Requires IL-7 and IL-15 for survival and quiescence.[2]Effector Memory T Cells (TEMs) Lack CD62L and CCR7(migrate to tissues), Rapid cytokine production upon reactivation.Derived from effector T cells, lose lymphoid homing markers but retain effector plasticity.[3]Tissue-Resident Memory T Cells (TRMs)Persist in non-lymphoid tissues, Express CD69 and CD103 for tissue retention.Imprinted by tissue-specific signals (e.g., TGF-β), Formed during immune response resolution.[4]
**B Cell Memory Subsets**
Classical Memory B CellsHigh-affinity BCRs due to somatic hypermutation, rapidly differentiate into plasma cells.Generated in germinal centers during T-dependent responses, Requires CD40-CD40L interactions.[5]Tissue-Resident Memory B CellsLocalized in peripheral tissues, Provide rapid local antibody responsesSustained antigen exposure or repeated stimulation in tissue niches enriched with survival factors.[6]

## 3. Memory T Cells in Infection

Memory T cells confer immunological memory by retaining antigen-specific receptors and functional adaptations from previous infections. Their rapid response upon antigen re-encounter ensures efficient pathogen clearance.

### 3.1. Central Memory T Cells (TCMs)

TCMs reside in secondary lymphoid organs and are responsible for maintaining a long-lived pool of memory T cells [7]. They are less differentiated than effector memory T cells (TEMs), possessing a high proliferative potential. Upon pathogen re-exposure, TCMs proliferate extensively and differentiate into effector T cells, which migrate to infection sites. This ensures a sustained immune response and replenishes effector and memory compartments. Studies have shown that TCMs play a vital role in long-term immunity against viral and bacterial pathogens, including *L. monocytogenes* and *M. tuberculosis*, by rapidly responding to re-infection due to their ability to circulate through the body and readily differentiate into effector T cells when encountering their specific antigen, effectively providing a sustained immune memory against these pathogens [8,9]. Interferon (IFN)-γ is crucial for the innate immune response against intracellular bacterial pathogens, traditionally attributed to natural killer (NK) cells. A previous study revealed that both effector and memory CD8+ T cells can secrete IFN-γ in response to interleukin (IL)-12 and IL-18, independent of antigen recognition. Memory CD8+ T cells specific for ovalbumin rapidly produce IFN-γ following infection with wild-type Listeria monocytogenes (LM) and significantly reduce bacterial loads in spleen and liver in IFN-γ–deficient mice. The upregulation of IL-12 and IL-18 receptors enables memory CD8+ T cells to respond in this antigen-independent manner, highlighting their pivotal role in innate immunity against intracellular pathogens [10]. In active pulmonary tuberculosis (TB), TCM-producing IFN-γ and TNF-α play a key role in mounting a protective immune response against *Mycobacterium tuberculosis* (Mtb). Antigenic stimulation increases TCM frequencies in healthy individuals, while TEMs predominate in active TB. In contrast, TCMs are more abundant in latent TB infection (LTBI). These shifts in memory T cell frequencies are closely linked to changes in antigen load, reflecting their dynamic response to infection status [11].

### 3.2. Effector Memory T Cells (TEMs)

TEMs circulate between blood and peripheral tissues and provide immediate effector functions such as cytokine production and cytotoxicity. Their rapid response is crucial for controlling infections at non-lymphoid sites. For example, during influenza virus reinfection, TEMs in the lung rapidly produce IFN-γ and recruit other immune cells to contain the virus [12]. TEMs are particularly important for controlling infections that require a swift immune response, such as malaria and *E. coli* [13]. TEMs express specific adhesion molecules that enable them to efficiently migrate to infected tissues, such as the liver in malaria and the intestinal mucosa in *E. coli* infections. Once in the infected tissue, TEMs can directly kill infected cells through cytotoxic mechanisms (e.g., releasing granzymes and perforin) or secrete cytokines to activate other immune cells and promote inflammation, helping to control pathogen replication. In malaria, TEMs are particularly important for controlling the liver stage of the parasite, where sporozoites initially infect hepatocytes. They can rapidly infiltrate the liver and eliminate infected cells, preventing further parasite dissemination. The role of circulating memory CD8+ T cells during liver-stage malaria infection has been clarified by findings that effector memory CD8+ TEMs infiltrate the liver within 6 h of malarial or bacterial infections and contribute to pathogen clearance. This rapid recruitment is accompanied by an upregulation of inflammatory genes in *Plasmodium*-infected livers and depends on TEM-intrinsic LFA-1 expression and liver phagocytes. These findings highlight the importance of TEMs in malaria protection and offer insights into mechanisms of liver infection control [14]. While less efficient against the blood stage of malaria due to the lack of MHC expression on infected red blood cells, TEMs can still contribute to immune control by activating other immune cells like macrophages to target parasitized erythrocytes.

### 3.3. Tissue-Resident Memory T Cells (TRMs)

TRMs are a distinct subset of memory T cells that reside permanently in barrier tissues, such as the skin, gut, and lungs, without recirculating. These cells act as frontline defenders by detecting and responding to pathogens at entry points [15]. TRMs have been shown to play a protective role in viral infections, such as herpes simplex virus (HSV) and influenza, by producing cytokines and enhancing local immunity [16,17]. Their presence in mucosal tissues also makes them critical for defense against respiratory and gastrointestinal infections. TRMs play a crucial role in controlling *Mycobacterium tuberculosis* (Mtb) infection. TRMs producing TNF-α, IL-2, and IL-17 proliferate rapidly in Mtb-infected lung tissues, and exogenous IL-17 or IL-2 enhance the anti-Mtb immune response [18]. Notably, BCG vaccination promotes long-term CD4+ TRM persistence in lung tissues, with these cells detectable for at least 12 months post-vaccination, underscoring their importance in sustained immunity against Mtb [8]. Although researchers hope to achieve long-term protection from TB by boosting TCM numbers using BCG and candidate vaccines, TCM numbers may decrease over time, due to chronic environmental Mtb exposure, which reduces TB vaccine efficacy. TRMs also play roles in chronic and persistent infections, where they help to limit the spread of pathogens, despite ongoing low-level infection. In chronic viral infections like HIV and hepatitis B, the persistence of TRMs in tissues such as the gastrointestinal tract and liver has been shown to impact the control of infection [19,20]. These cells can inhibit viral replication and prevent the reinfection of other tissues, although their function may be compromised in chronic settings due to immune exhaustion or chronic antigen exposure.

## 4. Memory T Cells in Autoimmunity

The immune system typically distinguishes between self and non-self to protect against pathogens while avoiding attacks on the body’s own tissues. However, in autoimmune diseases, this tolerance is lost, leading to an aberrant immune response against self-antigens. Autoimmune diseases, such as rheumatoid arthritis, multiple sclerosis, and type 1 diabetes, arise when self-reactive immune cells mistakenly target healthy tissues, resulting in chronic inflammation and tissue damage [21]. Memory T cells, which normally provide long-term protection against infections, can contribute to autoimmunity due to their persistence and heightened reactivity. Once primed against self-antigens, these cells can initiate and sustain chronic inflammatory responses, exacerbating disease progression. Unlike naïve T cells, memory T cells respond more rapidly and robustly upon antigen re-exposure, making them key players in autoimmune pathology. Their ability to resist immune regulation and persist long-term further complicates disease resolution. Understanding the mechanisms governing memory T cell involvement in autoimmunity is crucial for developing targeted therapeutic strategies.

### 4.1. Aberrant Activation of Memory T Cells

Memory T cells in autoimmune conditions are often characterized by heightened reactivity and resistance to regulatory mechanisms. For instance, in multiple sclerosis (MS), autoreactive memory CD4+ T cells specific for myelin antigens mediate central nervous system (CNS) inflammation [22]. Clinical studies have identified elevated frequencies of CD4+ and CD8+ memory T cells in the cerebrospinal fluid of MS patients, correlating with disease severity and relapse risk. Additionally, patients with relapsing–remitting MS show increased expression of CXCR3 and ICOS on memory T cells, which drive their migration and pathogenic function in CNS lesions [23,24]. When autoreactive memory T cells encounter their target antigen (myelin peptides in EAE) presented by antigen-presenting cells in the CNS, they quickly proliferate and differentiate into effector T cells, leading to a rapid inflammatory response. In experimental autoimmune encephalomyelitis (EAE), memory CD4+ T cells exhibit higher expression of activation markers, such as CD44, CXCR3, and ICOS, enhanced proliferation, and increased production of IFN-γ with reduced IL-17 secretion compared to effector T cells. Adoptive transfer of memory T cells into TCRαβ−/− recipients resulted in more severe disease, characterized by significant central nervous system inflammation and axonal damage [25]. Similarly, in type 1 diabetes, memory T cells targeting pancreatic islet antigens contribute to the destruction of beta cells [22]. A longitudinal study of T1D patients revealed a higher proportion of IFN-γ–producing CD4+ and CD8+ memory T cells in peripheral blood, correlating with C-peptide decline and disease progression. These findings suggest that therapeutic strategies targeting memory T cells may help preserve residual beta-cell function in early-stage T1D [26]. Memory T cells also demonstrate unique responses to immunomodulatory therapies. CTLA4Ig-mediated blockade of CD28-B7 signaling suppressed effector T cell-driven EAE but had little effect on memory T cell-induced disease. Conversely, ICOS-B7h blockade worsened effector T cell-mediated EAE but protected against memory T cell-induced disease. Blockade of the OX40 (CD134) costimulatory pathway ameliorated disease caused by both memory and effector T cells. These findings highlight the distinct pathogenic role of autoreactive memory T cells and provide insights for developing targeted therapies for autoimmune diseases [25].

### 4.2. Tissue-Resident Memory T Cells in Autoimmunity

TRMs, despite their protective roles in infection, can exacerbate tissue-specific autoimmune diseases. In psoriasis, for example, IL-17-producing TRMs persist in the skin and drive chronic inflammation [27]. A study analyzing psoriatic skin biopsies found that CD103+ CD8+ TRMs in resolved lesions retain the ability to produce IL-17A and IL-22 upon stimulation, explaining why psoriatic flares often occur at the same sites [28]. Despite effective treatments, psoriasis often recurs in previously affected areas, suggesting a site-specific disease memory. This study found enriched epidermal CD8 TRMs expressing CD103, CCR6, and IL-23R in resolved psoriasis. These cells produced IL-17A and CD4 T cells produced IL-22 upon stimulation, even after long-term TNF-α inhibition. These findings suggest that TRMs drive recurrent psoriasis by maintaining site-specific disease memory [7,27]. Likewise, in rheumatoid arthritis, TRMs in synovial tissue contribute to joint inflammation and damage [29]. Rheumatoid arthritis flares typically affect specific joints, driven by synovial TRMs. In murine models, CD8+ TRMs persist in previously inflamed joints during remission, showing long-term residency, fatty acid uptake, and limited migration. Upon activation, these TRMs recruit effector cells via CCL5, triggering flares. Depleting TRMs reduce site-specific recurrence. Similarly, human rheumatoid arthritis joints contain CD8+-dominant TRMs with pro-inflammatory signatures, suggesting TRMs as key drivers of disease chronicity and potential therapeutic targets [30]. A recent study analyzing colonic biopsies from IBD patients identified an enrichment of IFN-γ+ TRMs in inflamed mucosa, correlating with disease severity. Emerging therapies targeting gut TRMs, such as integrin inhibitors (vedolizumab) and sphingosine-1-phosphate (S1P) modulators (ozanimod), have shown efficacy in reducing inflammation by altering TRM migration and retention in the intestine [31].

### 4.3. Effector Memory T Cells and Chronic Inflammation

Due to their ability to persist in peripheral tissues and rapidly respond to re-exposure to self-antigens, TEMs play a key role in maintaining chronic inflammation in autoimmune diseases. TEMs, with their ability to migrate to inflamed tissues, are major players in systemic autoimmune diseases like systemic lupus erythematosus (SLE) [32]. Their production of inflammatory cytokines such as IFN-γ and TNF-α perpetuates tissue damage. Additionally, memory T cells longevity and capacity for clonal expansion sustain autoimmune responses over time. In RA, TEMs, particularly Th17 cells, have been shown to play a crucial role in the inflammation and joint destruction characteristic of the disease. These cells produce IL-17, which induces the production of pro-inflammatory cytokines and promotes the recruitment of other immune cells, exacerbating joint inflammation [33]. In addition, the persistence of TEMs in the synovium contributes to the chronicity of the disease. In multiple sclerosis (MS), TEMs, including both CD4+ and CD8+ T cells, are involved in the inflammatory response that leads to the destruction of the myelin sheath in the central nervous system (CNS). These cells are found in the cerebrospinal fluid of MS patients and in the affected lesions, where they promote inflammation and demyelination. The memory phenotype of these cells allows for rapid activation upon antigen re-exposure, contributing to recurrent relapses in MS [34].

## 5. Targeting Memory T Cells for Regulating Autoimmune Disease

Memory CD4+ T cells, including subsets such as Th1, Th2, Th17, and Tregs, are essential in the regulation of immune responses and play key roles in autoimmune diseases. Th1 and Th17 cells are known to drive inflammation and tissue damage, making them prime targets for therapeutic intervention. One approach to regulate these pathogenic T cells is through depletion strategies using monoclonal antibodies or other immune-modulating agents. For example, anti-CD4 antibodies have been tested in diseases like rheumatoid arthritis (RA) and multiple sclerosis (MS) to deplete activated CD4+ T cells, although challenges such as generalized immune suppression remain [35]. Targeting specific subsets like Th17 cells, which secrete pro-inflammatory cytokines such as IL-17, has shown success in diseases like RA and psoriasis. Drugs such as secukinumab, an anti-IL-17A antibody, have been approved for clinical use and are also being explored for their potential in targeting IL-23, which is crucial for Th17 cell maintenance [36]. Additionally, enhancing the function or expansion of regulatory T cells (Tregs), which are vital for maintaining immune tolerance, has been studied in conditions like systemic lupus erythematosus (SLE) and type 1 diabetes, with therapies such as low-dose IL-2 showing promise [37].

Targeting CD8+ T cells for depletion through monoclonal antibodies is one potential approach, particularly in diseases where these cells cause direct cytotoxicity. For instance, agents targeting surface markers like CD8 or inhibiting interactions between CD8+ T cells and their target cells may help reduce tissue damage [38]. Other strategies focus on modulating the activation of CD8+ T cells by targeting co-stimulatory molecules such as CD28 or CTLA-4, with the use of abatacept in RA demonstrating reduced CD8+ T cell activation and inflammation [39]. Additionally, an emerging approach involves inducing tolerance in autoreactive memory CD8+ T cells. This can be achieved by manipulating antigen presentation or administering tolerogenic vaccines or peptides, with promising results in preclinical models of type 1 diabetes [40].

## 6. Role of Memory B Cells in Infection

Memory B cells are pivotal in mounting rapid and effective immune responses during infections. Upon antigen re-encounter, these cells quickly proliferate and differentiate into plasma cells or secondary germinal center cells. Their roles are highlighted in the following infections:

Influenza: Memory B cells in the steady state generate a population of influenza-specific cells ready for rapid, enhanced antibody responses upon future exposure. While these antibodies are essential in preventing infections with the same influenza strain, they also exert immune pressure, selecting for drift variants that evade recognition by the host’s current influenza-specific antibody library. The influenza HA head has several distinct antigenic binding sites, and as mutations are selected within one site and, eventually, multiple sites, this creates gaps in the individual’s antibody recognition and neutralization of HA. Therefore, additional adaptive immune responses, such as T cell responses, are critical for optimal cross-protection against influenza [41,42].

SARS-CoV-2: Memory B cells persist after infection or vaccination, targeting spike protein epitopes. They play a role in long-term immunity and protect against severe COVID-19 upon re-infection [43]. A study of 254 COVID-19 patients over 8 months shows durable, broad-based immune responses to SARS-CoV-2. Spike-binding and neutralizing antibodies decay in two phases, with an extended half-life of over 200 days, indicating long-lived plasma cells. Infection also boosts antibody levels against SARS-CoV-1 and common betacoronaviruses. Spike-specific IgG+ memory B cells persist, supporting rapid antibody responses upon re-exposure or vaccination. Virus-specific CD4+ and CD8+ T cells are polyfunctional, with a half-life of 200 days. CD4+ T cells target multiple SARS-CoV-2 proteins, while CD8+ T cells preferentially target the nucleoprotein, suggesting its potential role in future vaccines [44].

The underlying causes of Long COVID-19 remain unclear, but emerging evidence suggests that persistent SARS-CoV-2 infection and the prolonged presence of spike protein in various tissues may drive chronic immune activation and inflammation [45]. Memory B cells may contribute to this process by sustaining immune responses against residual viral antigens, leading to prolonged inflammation and tissue damage. Additionally, dysregulated memory B cell responses, including the expansion of autoreactive B cell populations or persistent activation in the absence of active infection, have been implicated in post-viral syndromes and may play a role in the pathogenesis of Long COVID-19. Under normal conditions, memory B cell activation is tightly regulated to ensure inflammation subsides once the infection is cleared. However, in Long COVID-19, defective regulatory mechanisms may lead to prolonged cytokine production and sustained activation of other immune cells, such as T cells and macrophages. This ongoing immune stimulation could disrupt tissue homeostasis and contribute to multisystem dysfunction. Understanding the interplay between memory B cells, residual viral antigens, and immune dysregulation is critical for developing targeted therapies to mitigate Long COVID-19 symptoms.

Memory B cell responses can vary significantly between individuals due to several factors. Genetic differences, such as variations in immune-related genes, can influence the efficiency of memory B cell formation and longevity. Age also plays a role, with older individuals often experiencing weaker immune responses, leading to a more rapid decline in memory B cell function and reduced vaccine efficacy. Comorbidities like obesity, diabetes, and autoimmune diseases can impair memory B cell responses, while viral factors, such as antigenic variation in pathogens like influenza and COVID-19, can contribute to waning immunity and reinfection [46]. Environmental factors, including lifestyle choices and stress, further impact immune function and memory B cell durability. Additionally, vaccine-induced immunity varies depending on the platform and number of doses, with some individuals, particularly older adults, showing reduced longevity of memory B cell responses. Understanding these factors is essential for improving vaccine strategies and predicting immune responses across different populations.

HIV: Memory B cells are impaired in HIV infection due to chronic immune activation and viral replication. HIV primarily infects CD4+ T cells, leading to their depletion and progression to acquired immunodeficiency syndrome (AIDS). Although HIV does not directly infect B cells, it causes various perturbations in their function. B cells in HIV-infected individuals are hyperactivated, with elevated immunoglobulin secretion and hypergammaglobulinemia. However, they also show impaired responses to immunization and B cell receptor signals. Additionally, antigen-specific antibodies and memory B cell responses are reduced, compromising humoral immunity and increasing the risk of opportunistic infections and mortality [47]. HIV not only depletes CD4+ T cells but also impairs their function, even in individuals on effective antiretroviral therapy (ART) with undetectable viral RNA levels. Persistent immune dysfunction in these individuals can contribute to abnormal immune responses and increased susceptibility to infections. Despite the success of ART in suppressing viremia, the inability to fully restore immune function remains a major challenge. Broadly neutralizing antibodies (bNAbs), which prevent viral entry by targeting conserved regions across various HIV strains, are a key focus in HIV vaccine design. While traditional vaccines have struggled to elicit these antibodies, some individuals with chronic HIV infection develop potent bNAbs [48]. However, HIV’s high mutation rate, glycan shielding, and ability to establish latent reservoirs have made vaccine development particularly challenging. Alternative strategies are being explored to develop effective HIV vaccines, including the following:

Germline-targeting vaccine approaches—Designed to guide B cell maturation toward bNAb production by sequential immunization.
mRNA-based HIV vaccines—Leveraging recent advances in mRNA technology to improve immunogenicity and antibody response.
T cell-based vaccines—Aiming to induce strong CD8+ T cell responses that could help control viral replication.
Latency-reversing agents combined with immunotherapy—Strategies aimed at exposing and eliminating latent HIV reservoirs.

### 6.1. Effector Functions in Bacterial Infections

Memory B cells specific to *Salmonella typhi* can persist for years and can rapidly differentiate into plasma cells upon reinfection, producing high-affinity antibodies that neutralize the pathogen more efficiently than during the primary infection. These antibodies are crucial for opsonizing the bacteria and facilitating their clearance through enhanced phagocytosis by macrophages and neutrophils. Moreover, memory B cells can help in the formation of germinal centers upon secondary exposure, enabling the generation of higher-affinity antibodies through somatic hypermutation and class-switch recombination. Studies have shown that in Salmonella typhi-infected individuals, the presence of memory B cells contributes to faster clearance of the bacteria upon reinfection, highlighting their importance in long-term immunity and protection against the pathogen [48]. Effective vaccines also generate long-lived memory B cells, which can quickly respond to re-infection and produce higher affinity antibodies due to somatic hypermutation, often requiring multiple doses to boost antibody affinity. These responses prevent bacteremia and systemic spread, ensuring long-term protection [49].

### 6.2. Role of Memory B Cells in Chronic Infections

Tuberculosis (TB): While the primary focus in TB vaccine development has historically been on stimulating T cell responses, recent studies indicate that B cells and their antibodies play a more significant role in controlling *M. tuberculosis* infection than previously thought. Antibodies produced by memory B cells can opsonize M. tuberculosis bacteria, facilitating their phagocytosis by macrophages, and may also contribute to antibody-dependent cell-mediated cytotoxicity (ADCC) where immune cells kill infected cells coated with antibodies [50].

Hepatitis B Virus (HBV): HBV vaccines generate long-lived memory B cells that ensure protection even after antibody levels wane. Booster doses rapidly reactivate these cells, highlighting their role in maintaining immunity. It is widely recognized that many vaccinated individuals, especially those immunized in childhood, experience a decline in anti-HBs titers below protective levels. However, individuals with protective anti-HBs titers at the time of vaccination show a rapid anamnestic response when boosted, indicating that memory immunity is crucial for protection against clinical disease and the development of a carrier state, regardless of antibody levels. Even in the absence of anti-HBs, significant amounts of HBsAg-specific memory T and B cells are present in vaccine responders. As a result, booster doses are not recommended for individuals with normal immune status, despite the decline in anti-HBs titers, though this may be a concern for high-risk groups [51].

Tissue-Resident Memory B Cells in Barrier Defense—Memory B cells residing in tissues such as the lungs and gut provide localized defense. Tissue-resident memory B cells in the lungs rapidly secrete IgA and IgG upon encountering respiratory pathogens like influenza and RSV, effectively neutralizing these viruses by binding to their surface antigens, thereby preventing them from infecting host cells and providing a crucial first line of defense against reinfection in the respiratory tract. By maintaining a resident population of virus-specific B cells in the lungs, the body is prepared to rapidly respond to subsequent exposures to the same pathogen, potentially preventing severe disease [52]. Gut memory B cells play a vital role in the immune response to specific infections, particularly those affecting the gastrointestinal tract. For example, during *Clostridium difficile* infection, gut memory B cells are critical for producing IgA antibodies that help prevent pathogen adherence and toxin production in the gut. Following an initial infection, memory B cells specific to *C. difficile* can quickly respond upon re-exposure, offering protection from reinfection [53]. Gut memory B cells are key in defending against rotavirus, a leading cause of gastroenteritis in children. After an initial infection or vaccination, these cells generate long-lasting IgA antibodies, which are secreted into the intestines to neutralize the virus and prevent further infection [54]. During *Salmonella* infections, gut memory B cells help produce antibodies that neutralize the bacteria and prevent systemic spread. These cells persist in the gut lymphoid tissues and rapidly respond upon subsequent exposure to *Salmonella* [55].

### 6.3. Memory B Cells in Autoimmune Diseases

Memory B cells, which are typically involved in providing long-term immunity against pathogens, can become dysregulated in autoimmune conditions, contributing to chronic inflammation and tissue damage. Below are several autoimmune diseases where memory B cells have been implicated:

Systemic Lupus Erythematosus (SLE): Memory B cells play a pivotal role in the pathogenesis and progression of systemic lupus erythematosus (SLE), a chronic autoimmune disease characterized by the production of autoantibodies and systemic inflammation. These cells are a primary source of autoantibodies targeting nuclear antigens, such as double-stranded DNA (dsDNA) and ribonucleoproteins. The autoantibodies form immune complexes that deposit in tissues, triggering inflammation, complement activation, and subsequent organ damage. In SLE patients, memory B cells often exhibit abnormal phenotypes and dysregulated functions. They are hyperactivated due to aberrant signaling through their B cell receptors (BCRs) and Toll-like receptors (TLRs), particularly TLR7 and TLR9 [56]. This overactivation contributes to an expansion of atypical memory B cells, including age-associated B cells (ABCs), which are resistant to apoptosis and perpetuate chronic immune activation. Moreover, these memory B cells display increased expression of co-stimulatory molecules, enhancing their interactions with T follicular helper (Tfh) cells in germinal centers. This promotes the survival, proliferation, and differentiation of autoreactive B cells into long-lived plasma cells that continuously secrete pathogenic autoantibodies. Memory B cells in SLE also show reduced thresholds for activation, leading to their heightened responsiveness to immune stimuli. Collectively, these factors position memory B cells as central players in driving the autoimmune processes and chronic inflammation characteristic of SLE [57].

Rheumatoid Arthritis (RA): Memory B cells play a pivotal role in the pathogenesis of rheumatoid arthritis (RA), particularly in sustaining chronic inflammation and autoimmunity. In RA, memory B cells are implicated in the production of autoantibodies, such as rheumatoid factor (RF) and anti-citrullinated protein antibodies (ACPAs). These autoantibodies target self-antigens, which are thought to contribute to the inflammatory processes in the joints. For instance, ACPA, which targets citrullinated proteins, has been linked to the development of RA and the progression of joint damage. Moreover, memory B cells in RA are not just involved in antibody production; they also participate in the persistence of disease by acting as a source of pro-inflammatory cytokines. These cytokines can help recruit and activate other immune cells in the joints, sustaining the inflammatory environment [58].

Multiple Sclerosis (MS): Memory B cells are crucial players in the immune response and have a significant role in the pathogenesis of MS, an autoimmune disease of the central nervous system (CNS). In MS, memory B cells contribute to disease development and progression by producing autoantibodies and promoting the inflammatory cascade within the CNS. When activated, memory B cells differentiate into plasma cells, which secrete autoantibodies that can bind to myelin proteins, such as myelin oligodendrocyte glycoprotein (MOG) and proteolipid protein (PLP). These autoantibodies can form immune complexes that contribute to inflammation and damage in the CNS. This damage is primarily driven by the activation of microglia and the recruitment of other immune cells, such as T cells, which exacerbate the inflammatory response. The damage to the myelin sheath leads to demyelination, impaired nerve conduction, and the neurological symptoms characteristic of MS, including muscle weakness, vision problems, and cognitive dysfunction. In addition to antibody production, memory B cells in MS also contribute to the disease through their ability to present antigens and secrete pro-inflammatory cytokines. These cytokines can further enhance the activation of T cells, creating a feedback loop that sustains the inflammatory environment in the CNS. Memory B cells have been found in the cerebrospinal fluid (CSF) and lesions of MS patients, providing further evidence of their involvement in the disease process [59].

Graves’ Disease: In Graves’ disease, memory B cells are thought to be activated by the recognition of self-antigens, such as the TSH receptor, which is expressed on the surface of thyroid cells. Upon activation, these memory B cells differentiate into plasma cells that produce autoantibodies against the TSH receptor. These autoantibodies mimic the action of TSH, overstimulating the thyroid gland and resulting in excessive secretion of thyroid hormones, including thyroxine (T4) and triiodothyronine (T3), which leads to the symptoms of hyperthyroidism, including weight loss, rapid heart rate, and tremors [60]. Moreover, memory B cells in Graves’ disease are involved in sustaining the autoimmune response. They can circulate in the peripheral blood, and when re-exposed to thyroid antigens, they rapidly produce large amounts of autoantibodies, which continue to stimulate the thyroid gland. This mechanism of memory B cell activation is one of the reasons why Graves’ disease tends to be chronic, with periods of flare-ups and remissions. The continued presence of memory B cells in the circulation helps maintain the autoimmune response and contributes to the relapsing nature of the disease [60].

Myasthenia Gravis (MG): Memory B cells play a pivotal role in the autoimmune disorder Myasthenia Gravis (MG), a condition characterized by muscle weakness due to impaired neuromuscular transmission. In MG, the immune system produces autoantibodies that target and block the acetylcholine receptors (AChRs) on the postsynaptic membrane of neuromuscular junctions. These autoantibodies hinder the normal communication between nerve and muscle cells, leading to muscle weakness. Memory B cells are central to the persistence of this autoimmune response, as they produce and maintain the production of these autoantibodies, which are a hallmark of MG [53].

Hashimoto’s Thyroiditis: Memory B cells in Hashimoto’s thyroiditis continue to produce autoantibodies against thyroid antigens, leading to progressive damage to the thyroid gland. This dysregulation of B cell responses is central to the chronic autoimmune process in the disease [53,54].

Autoimmune Hemolytic Anemia (AIHA): Memory B cells are responsible for the chronic production of autoantibodies that target red blood cells, leading to their destruction and resulting in anemia. These cells persist and can be reactivated, contributing to the chronic nature of the disease [61].

Systemic Sclerosis (SSc): A study showed that memory B cells in SSc patients produce autoantibodies against topoisomerase I and centromere proteins, which are associated with the pathogenesis of the disease [62]. These autoantibodies are considered biomarkers for disease subtypes and prognosis.

## 7. Targeting Memory B Cells for Regulating Autoimmune Disease

Given their pathogenic role, targeting memory B cells has emerged as a promising therapeutic strategy to regulate autoimmune responses while sparing naïve B cells, which are essential for maintaining normal immune function. These strategies focus on dampening the activity or promoting tolerance of autoreactive immune cells.

### 7.1. Depleting Memory B Cells

Therapies like rituximab, a monoclonal antibody targeting CD20, can deplete B cells, including subsets of memory B cells. Although CD20 is not expressed on plasma cells, which produce antibodies, targeting CD20 can effectively reduce the pool of precursor memory B cells, leading to reduced autoantibody production over time. For example, in SLE and RA, rituximab has shown efficacy in reducing disease activity and delaying flares. However, the incomplete depletion of memory B cells and the persistence of autoantibody-secreting plasma cells remain challenges [55]. Additionally, emerging therapies like Janus kinase inhibitors (JAK inhibitors), which target intracellular signaling pathways involved in both memory B and T cell activation, are being evaluated in clinical trials for diseases like SLE and RA, with some showing efficacy in reducing disease activity. However, these treatments come with potential side effects, including increased risks of thrombosis, malignancies, and infections. While these therapies provide substantial benefits for many patients, their side effect profiles and long-term impacts on immune function require ongoing evaluation in clinical settings.

### 7.2. Blocking Survival and Reactivation Signals

Memory B cells rely on survival signals provided by cytokines such as BAFF (B-cell activating factor) and APRIL (a proliferation-inducing ligand). Drugs like belimumab, a BAFF inhibitor, reduce the survival of autoreactive B cells, including memory B cells, and have been approved for SLE treatment [63]. Additionally, disrupting interactions between memory B cells and T cells via co-stimulatory molecules, such as CD40 or ICOS, can prevent the reactivation of memory B cells, reducing their pathogenic contributions [64].

Methodology: To provide a comprehensive and balanced review, we selected studies based on relevance to the topic of memory T cells in infection and autoimmunity. We included both recent and foundational studies, ensuring that key discoveries and evolving perspectives were represented. Priority was given to peer-reviewed primary research and high-impact reviews, with a focus on mechanistic insights and clinical relevance

## 8. Conclusions

Memory cells play a central role in autoimmune diseases, and targeting these cells holds therapeutic promise. Future research should focus on developing therapies that selectively modulate autoreactive memory B and T cells while preserving protective immunity. Understanding how memory cells interact with the tissue microenvironment and exploring combination therapies with cytokine inhibitors or gene editing could enhance therapeutic efficacy. Additionally, longitudinal studies are needed to assess the long-term effects of these therapies on immune memory and overall health. However, several challenges remain in the clinical implementation of memory-cell-targeted therapies. Off-target effects, variability in patient responses, and the risk of impairing protective immunity are key concerns. The cost and accessibility of such therapies, particularly in low-resource settings, also pose significant barriers. Overcoming these challenges will require a collaborative approach among immunologists, clinicians, and regulatory bodies to ensure the safe and effective application of these therapies in autoimmune disease management.

## Data Availability

No new data were created or analyzed in this study. Data sharing is not applicable to this article.
